# Development of *in vitro* and *in vivo* rabies virus neutralization assays based on a high-titer pseudovirus system

**DOI:** 10.1038/srep42769

**Published:** 2017-02-20

**Authors:** Jianhui Nie, Xiaohong Wu, Jian Ma, Shouchun Cao, Weijin Huang, Qiang Liu, Xuguang Li, Yuhua Li, Youchun Wang

**Affiliations:** 1 Division of HIV/AIDS and Sex-transmitted Virus Vaccines, National Institutes for Food and Drug Control (NIFDC), No. 2 Tiantanxili, Beijing 100050, China; 2 Division of Arboviral Vaccines, National Institutes for Food and Drug Control (NIFDC), No. 2 Tiantanxili, Beijing 100050, China; 3 Centre for Biologics Evaluation, Biologics and Genetic Therapies Directorate, Health Canada, Ottawa, On K1A 0K9, Canada; 4 Department of Biochemistry, Microbiology and Immunology, Faculty of Medicine, University of Ottawa, On, Canada

## Abstract

Pseudoviruses are useful virological tools because of their safety and versatility; however the low titer of these viruses substantially limits their wider applications. We developed a highly efficient pseudovirus production system capable of yielding 100 times more rabies pseudovirus than the traditional method. Employing the high-titer pseudoviruses, we have developed robust *in vitro* and *in vivo* neutralization assays for the evaluation of rabies vaccine, which traditionally relies on live-virus based assays. Compared with current rapid fluorescent focus inhibition test (RFFIT), our *in vitro* pseudovirus-based neutralization assay (PBNA) is much less labor-intensive while demonstrating better reproducibility. Moreover, the *in vivo* PBNA assay was also found to be superior to the live virus based assay. Following intravenous administration, the pseudovirus effectively infected the mice, with dynamic viral distributions being sequentially observed in spleen, liver and brain. Furthermore, data from *in vivo* PBNA showed great agreement with those generated from the live virus model but with the experimental time significantly reduced from 2 weeks to 3 days. Taken together, the effective pseudovirus production system facilitated the development of novel PBNA assays which could replace live virus-based traditional assays due to its safety, rapidity, reproducibility and high throughput capacity.

Rabies remains an acute zoonotic disease with a case-fatality rate of approaching 100%, causing almost 60,000 deaths annually^1^,^2^; the majority of cases are found in Asia and Africa^3^,^4^. Although a lethal disease, rabies could be effectively prevented by post-exposure prophylaxis (PEP) regimen. Specifically, prompt administration of vaccines in conjunction with rabies-immunoglobulins and proper wound management after exposure prevent rabies even after high-risk exposure^4^,^5^. Animal studies have demonstrated that rabies-specific antibodies, especially the neutralizing antibodies, played an essential role in vaccine-mediated protection^6^. Yet, clinical evaluation of rabies vaccines in humans is not possible due to ethical considerations. Therefore, new rabies vaccines or rabies-specific immunoglobulins must be evaluated for their potency by *in vitro* and *in vivo* assays prior to being authorized for human use^7^.

The current assays for immunogenicity determination of rabies vaccines and immunoglobulin have several inherent limitations. Specifically, vaccine-induced or natural infection-elicited antibody responses against rabies virus are determined using serological assays including the rapid fluorescent focus inhibition test (RFFIT)^8^, fluorescent antibody virus neutralization (FAVN) test^9^ and enzyme linked immunosorbant assay (ELISA)^10^. Currently, the “gold standard” for *in vitro* assays are RFFIT and FAVN, both of which are routinely used in WHO reference laboratories; however, both assays require the use of live rabies viruses which must be handled in biosafety level 2 (BSL-2) containment facilities^8^,^9^,^11^. Although inactivated viruses are used as coating antigens in ELISA, the assay cannot be used to assess neutralizing antibodies^10^. While the *in vitro* neutralization assay is used to determine if rabies vaccine could induce neutralizing antibodies in animals, results generated from the *in vitro* neutralizing assays are not always correlated to those from *in vivo* protection^12^,^13^. Currently, a variety of animal models have been used for the evaluation of new rabies vaccines^4^,^12^,^14^,^15^. However, live viruses must still be used to challenge the animals immunized with the vaccines in the animal biosafety level 2 (ABSL-2) facilities^11^. Taken together, alternative assays without the aforementioned limitations should be explored.

Pseudovirus has been widely used in place of the corresponding live virus in serological screening, vaccine efficacy assessment, gene transduction and other virological studies. In a virus or pseudovirus, the capsid encapsulating the RNA genome is the “core” while the outer membrane functions as the “envelope”, which determines the tropism of the virus or pseudovirus. Pseudoviruses have been successfully used in the studies of a variety of enveloped viruses such as Ebola virus^16^, Middle Eastern Respiratory syndrome (MERS) virus^17^, hepatitis C virus^18^, influenza virus^19^. However, technical challenges have been encountered by investigators in making high-titer pseudoviruses. Specifically, pseudotyping of lentiviral vectors with RABV glycoprotein G has been explored in experimental gene therapy against neurological disorders^20^,^21^,^22^ and determination of neutralizing antibody^23^,^24^; however, low transduction efficiency associated with the glycoprotein G pseudotyped virus substantially hinders its wider applications^25^,^26^. This is especially true for *in vivo* animal studies, in which no rabies pseudovirus has been reported. While it is likely that chimeric glycoproteins with VSV-G could partially resolve transduction issue^27^, the use of chimeric glycoprotein would compromise the application of such pseudovirus to the analyses of neutralizing antibody targeting the envelope of the wild type virus. Clearly, novel approaches should be explored to circumvent these technical difficulties.

In this communication, we present a novel platform capable of generating high-titer rabies pseudovirus, which are of high quality for the development of novel pseudovirus-based neutralizing assays (PBNA) for both *in vitro* and *in vivo* analyses of rabies-specific immunoglobulin and vaccines-induced immunogenicity. We demonstrated here that the pseudovirus-based assays are superior to the current assays in terms of reproducibility, and safety. The turn-around time for the pseudovirus *in vivo* assay was shorter than the original virus assay. The optimization of the procedure reported here could greatly facilitate development of similar assays to replace other highly pathogenic enveloped viruses for the assessment of wider range of vaccines and gene therapy products.

## Results

### Construction and optimization of rabies G protein and HIV-1 backbone expressing plasmids

To compare the efficiency of different promoters, firefly luciferase (*fluc*) gene was cloned into pcDNA3.1 to generate pcDNA3.1.fluc, which is used in this study as a control. Three additional *fluc* expressing plasmids containing different promoters were then constructed by replacing the CMV promoter (cCMV) of the pcDNA3.1.fluc with complete CMV promoter (sCMV), CAG or LTR to generate psCMV.fluc, pCAG.fluc or pLTR.fluc, respectively. 293T cells were transfected with the aforementioned four plasmids, with relative light units (RLU) being analyzed 48 hours post-transfection. Demonstrated by the highest RLU values, the construct psCMV.fluc carrying the complete CMV promotor were found to be most effective in driving gene expression.

It is noted that pNL4-3.luc is widely used to make pseudotyped virus; however, the low titers of the pseudoviruses make them unsuitable for a lot of other experiments, particularly *in vivo* assays which require viral titers being at least 10^6^ TU/ml. To improve the titer of pseudovirus, we introduced pSG3ΔEnv into the rabies pseudotyped virus. pSG3ΔEnv was previously used as the backbone plasmid in a validated HIV neutralization assay, in which high titers of pseudotyped viruses were generated^28^,^29^,^30^. However, the pSG3ΔEnv could not be directly used to make rabies G-pseudotyped virus as it lacks the reporter gene in the backbone plasmid. Therefore, we cloned *fluc* gene into this pSG3ΔEnv at the end of the silenced *env* gene to generate pSG3ΔEnv.fluc.

We next set out to determine the optimal pairing of G protein expressing plasmid with different backbone plasmids. *fluc* genes were replaced with CVS glycoprotein gene in the aforementioned fluc expressing plasmids with different promoters to generate pcDNA3.1.CVS (3.1), psCMV.CVS (C), pCAG.CVS (A), and pLTR.CVS (L) respectively. To generate various forms of pseudoviruses, pNL4-3.luc (N), pSG3ΔEnv.fluc (F) or pSG3ΔEnv.sCMV.fluc (FC) were co-transfected separately with the four G protein expression plasmids into 293T cells; the twelve forms of pseudotyped viruses generated as such were subsequently used to infect 293T cells; forty-eight hours post-infection, RLU was determined. We found that the pairing pCAG.CVS with pSG3ΔEnv.sCMV.fluc could produce the highest RLU values (data not shown).

To optimize the conditions for the generation of pCAG.CVS-pSG3ΔEnv.sCMV.fluc, we tested the ratio between pCAG.CVS and pSG3ΔEnv.sCMV.fluc. To this end, ratios of pCAG.CVS to pSG3ΔEnv.sCMV.fluc ranging from 1:6 to 6:1 were investigated; we found the 3:1 ratio is ideal to yield the highest titer of pseudotyped virus, with TCID_50_ reaching as high as 1 × 10^7^ TCID_50_/ml.

### Optimization of cell type and number

**Figure 1:**
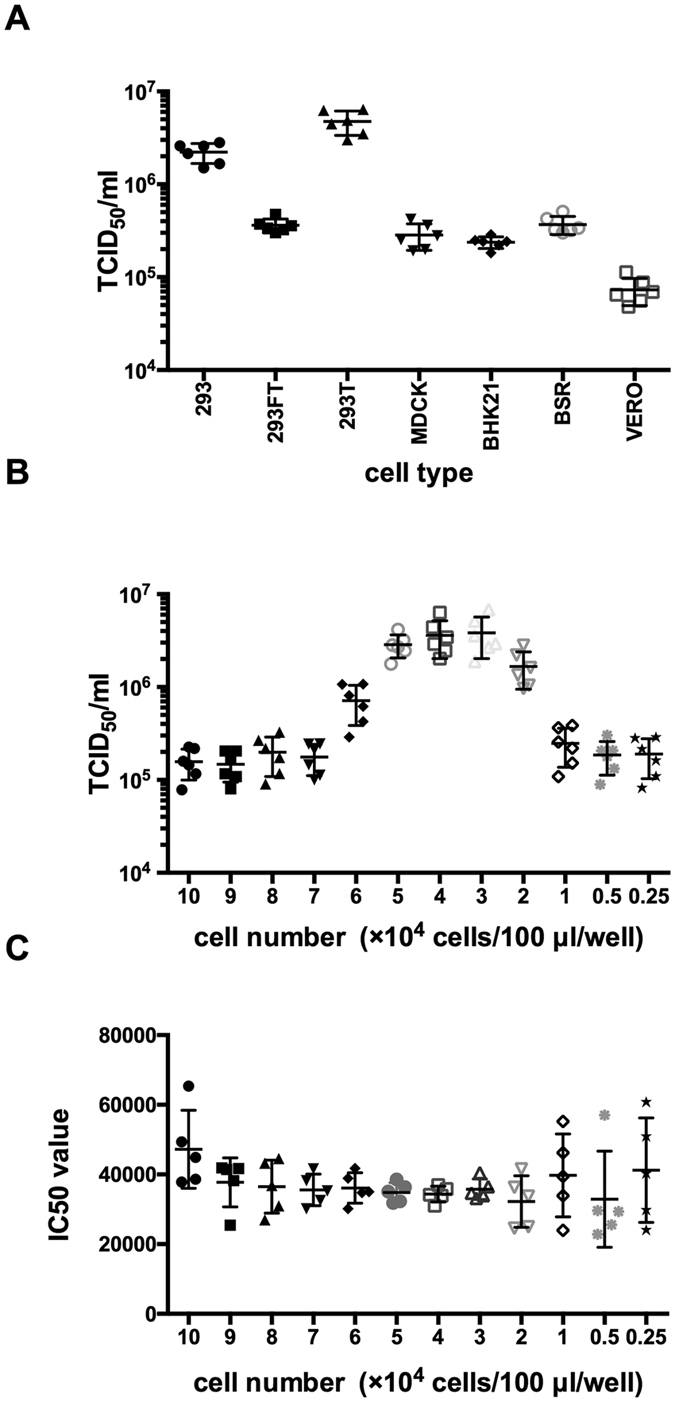
Optimization of the cells used in the *in vitro* rabies pseudovirus neutralization assay. Panel A: the the selection of the cell line. The same lot of rabies pseudoviruses were titrated six times in each cell lines. The highest pseudovirus titers were obtained in 293T cells. Panel 1B:effect of the cell number on pseudovirus titration. For 293T cells, rabies pseudotyped virus were titrated over a range of cell numbers (2,500 to 100,000/well). 40,000/well was found to yield the highest titer when equal concentration of virus preparations was used. Panel 1 C: effect of cell number on pseudovirus neutralization assay. The values of 50% maximal inhibitory concentration (IC_50_) values were similar to those with cell inocula ranging from 10,000 to 80,000/well. When the input cells numbers were more than 50,000/well or less than 30,000/well, the variation of the IC_50_ values would increase dramatically.

Having found pCAG.CVS and pSG3ΔEnv.sCMV.fluc was the best combination, we next investigated the cell types in generating rabies pseudovirus. To achieve this, seven types of cells were tested, including HEK293, 293T, 293FT, MDCK, BHK21, BSR and Vero. The titers of the rabies pseudovirus generated in different cell lines were compared using one-way analysis of variance (ANOVA) and showed significant difference (n = 6, p < 0.001) (Fig. 1A). 293T cells were the best cell substrate for pseudovirus neutralization assay.

We next determined the optimal numbers of 293T cells for rabies virus production. Towards this end, we titrated the rabies pseudotyped virus at a wide range of cell numbers (2,500 to 100,000/well). 40,000/well was found to yield the highest titer when equal concentration of virus preparations were used (Fig. 1B). The titers of the rabies pseudovirus detected at different cell numbers were compared using one-way ANOVA and showed significant difference (n = 6, p < 0.001). Furthermore, when paired comparisons (student’s t test) were performed, no significant difference (p > 0.05) was observed between 40,000/well and 30,000/well, while the difference between 40,000/well and 50,000/well was also found to be insignificant. It is of note that the linear correlation coefficients (R) obtained at cell inoculum from 10,000 to 50,000/well were found to be greater than 0.99, revealing an excellent linear curve fitting. Moreover, when neutralization assays were performed with different cell numbers, the values of 50% maximal inhibitory concentration (IC_50_) values were similar to those with cell inocula ranging from 10,000 to 80,000/well (Fig. 1C). However, when the input cells numbers were more than 50,000/well or less than 30,000/well, the variation of the IC_50_ values would increase dramatically. Given these findings, 40,000/well was chosen for subsequent experiments.

### Optimization of virus dose for neutralization assay

**Figure 2:**
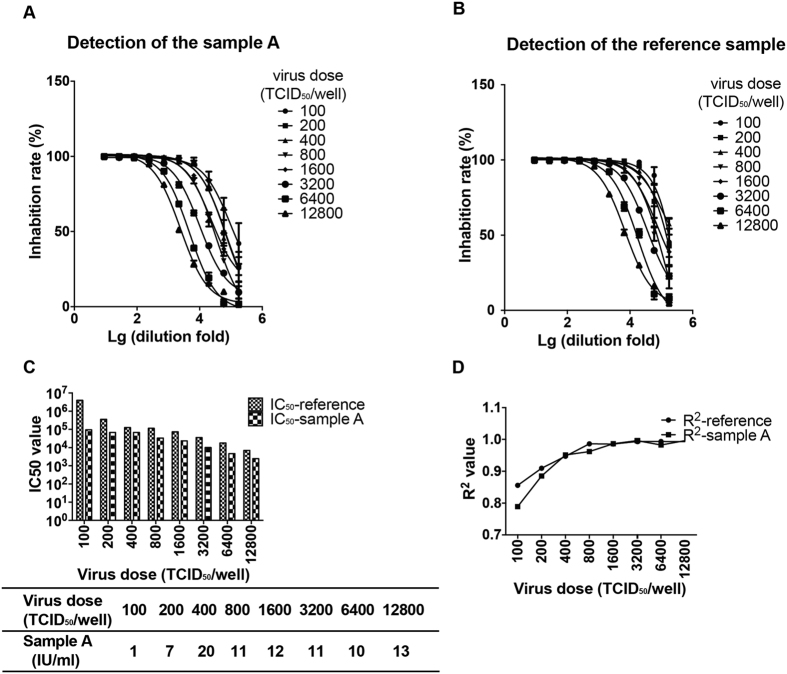
Optimization of virus dose for neutralization. Panel 2 A: effect of the pseudovirus dose on the neutralization curve for the human serum sample. The absolute IC_50_ values decreased gradually with the increasing amounts of the viral inocula Panel 2B: effect of the pseudovirus dose on the neutralization curve for the national standard. Panel 2 C: effect of the pseudovirus dose on value assignment for sample A. When the value of sample A was assigned against the reference sample, the titers fell within the acceptable range with virus inocula ranging from 800 to 12800 TCID_50_/well. Panel 2D: effect of the pseudovirus dose on the R^2^ values for sample A and national standard. Lower viral inocula resulted in larger variability and less curve fitting (smaller R^2^ values).

We next tested the viral inocula with a dose range starting from 100 to 12800 TCID_50_/well for the *in vitro* neutralization assay. The Chinese national reference sample (37.0 IU/ml) and an internal control sample (designated as sample A, 7.1–12.9 IU/ml) for RFFIT assay were employed for viral dose optimization. As expected, the absolute IC_50_ values decreased gradually with the increasing amounts of the viral inocula (Fig. 2A,B and C). Furthermore, when the value of sample A was assigned against the reference sample, the titers fell within the acceptable range with virus inocula ranging from 800 to 12800 TCID_50_/well. It is noted that lower viral inocula resulted in larger variability and less curve fitting (smaller R^2^ values). Specifically, although the titer of sample A also met the acceptable criteria with lower viral inoculum (200 TCID_50_/well), there is a trend of increasing variability if the viral inocula were less than 400 TCID_50_/well (Fig. 2A and B), as further supported by decreasing R^2^ values. Therefore, we chose the viral inocula at 1600 TCID_50_/well as the optimal dose.

### Optimization of DEAE-dextran concentration for pseudovirus infection

As part of optimization process, we also determined DEAE-dextran concentrations ranging from 0 to 60 μg/ml. RLU values were found to increase with the increasing amounts of DEAE-dextran between 0 to 7.5 μg/ml; the RLU readings plateaued with the DEAE-dextran concentrations between 7.5 to 15 μg/ml before they decreased sharply with the concentration of DEAE-dextran reaching over 30 μg/ml. The decreased RLU values were found to be due to cellular toxicity induced by DEAE-dextran; specifically, the viability of the cells decreased when DEAE-dextran concentration reached 15 μg/ml or higher.

In the neutralization assay, although the addition of DEAE-dextran could reduce the virus volume to yield similar levels of SNR, this had little effect on the final results. Based on these observations, 10 μg/ml of DEAE-dextran was chosen to enhance the infectiousness of the pseudovirus without causing cytotoxicity.

### Sensitivity, reproducibility and correlation of the PBNA with the RFFIT

As the pseudovirus-based neutralizing assay (PBNA) assay is intended to be used for the analyses of both human and animal samples, a panel of fifty human and thirty mouse serum samples were used to evaluate the PBNA in parallel with RFFIT for comparison.

**Figure 3:**
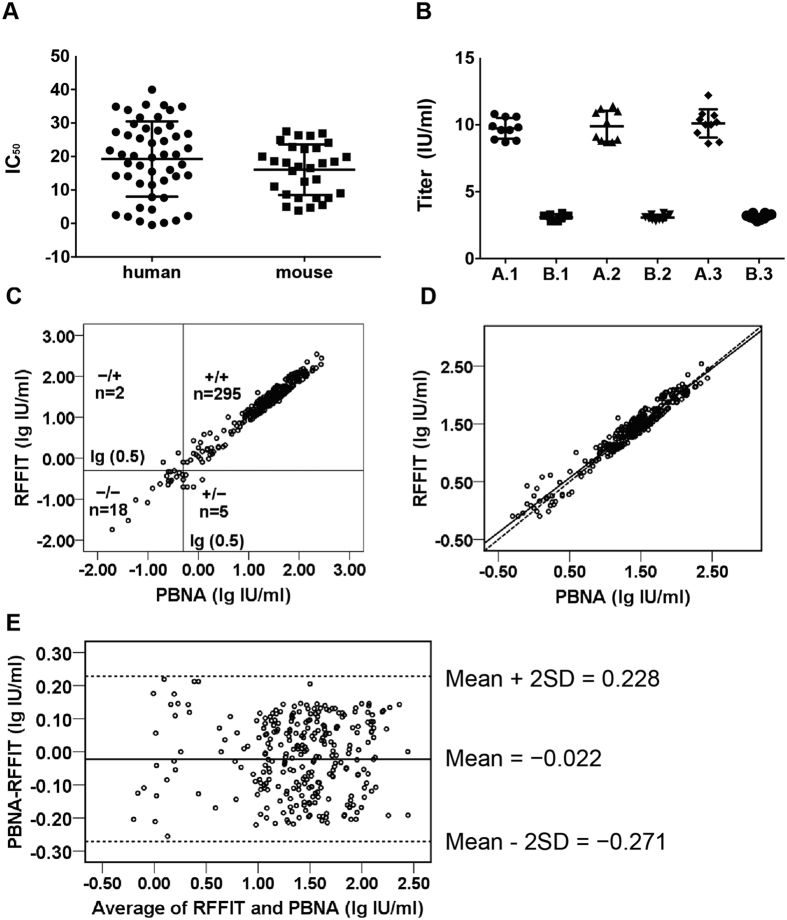
Evaluation of PBNA. Panel A: determination of cut-off vaule in PBNA. A panel of fifty negative human and thirty mouse serum samples were used to determine the limit of detection (LOD). LOD was 0.015 IU/ml and 0.011 IU/ml for the human and mouse serum samples, respectively. Panel B: reproducibilty. The reproducibility of the PBNA was determined by testing the two internal control samples in three independent runs, with each run the two samples being tested 3 times on 10 individual plates. The coefficient of variation (CV) ranged from 6.7% to 11.7% for the intra assay and 6.6% to 9.9% for the inter assay. Panel 3 C: correlation between PBNA and RFFT forcomparison of the PBNA and RFFIT for all the 320 samples. The qualitative comparison of the results from the two assays showed concordance of 295 positive and 18 negative samples (97.81%, kappa = 0.826), respectively. Panel 3D: correlation between PBNA and RFFT in relation tolinear relationship for 295 positive samples. The fitted regression line is presented by the equation PBNA = 0.998 × RTTIT −0.019, R^2^ = 0.946. The solid line represents the fitted regression line and the dashed line represents a perfect match between the two assays. Panel 3E: agreement between the PBNA and RFFIT tests for 295 positive samples analyzed by Bland-Altman plot.

We first established the limit of detection (LOD) for PBNA assay. As shown in Fig. 3A, when assayed by PBNA, human serum samples were found to have higher background compared to the mouse samples. When the mean titer value of the negative samples +1.96 standard deviation (SD) was used to calculate the limit of detection (LOD), the LOD was 38.7 for human serum samples and 27.9 for mouse serum samples. When compared with the national anti-rabies standard (37.0 IU/ml), the LOD was 0.015 IU/ml and 0.011 IU/ml for the human and mouse serum samples, respectively.

The values for the two internal control samples were initially assigned using the RFFIT (testing 7 times on 5 individual plates) 7.1–12.9 IU/ml for A and 2.2–3.6 IU/ml for B by mean ± SD. The reproducibility of the PBNA was determined by testing the two internal control samples in three independent runs, with each run the two samples being tested 3 times on 10 individual plate (each in duplicate wells). The coefficient of variation (CV) ranged from 6.7% to 11.7% for the intra assay and 6.6% to 9.9% for the inter assay (Fig. 3B). The total CVs were 6.4% and 10.1% for sample A and B in PBNA respectively, which were relatively lower than those in RFFIT (29.0% and 24.1%). All results tested by PBNA were within the assigned values pre-determined by RFFIT. Based on results generated by PBNA (30 tests), value ranges were assigned for sample A (8.9–10.9 IU/ml) and B (2.9–3.3 IU/ml).

To further determine the correlation between the PBNA and RFFIT in qualitative analyses of rabies-specific antibodies, 320 serum samples from vaccinated human subjects were assayed. Among the 320 clinical samples, 297 (92.81%) were identified as positive by RFFIT and 300 (93.75%) by PBNA assays, respectively (Fig. 3C). The qualitative comparison of the results from the two assays showed concordance of 295 positive and 18 negative samples (97.81%, kappa = 0.826), respectively, with discordance being found for seven samples only (McNemar’s *p* = 0.453). Further analysis revealed that the neutralizing antibody values of these seven samples were determined as 0.6, 0.5, 0.8, 1.2, 0.5 by PBNA, and 1.3, 0.8 by RFFIT; most of these seven values were close to the cutoff value (0.5 IU/ml), indicating discordance was mainly associated with serum samples with very low levels of antibodies, i.e., near the borderline set for positive value.

Correlation between the two assays was also determined in quantitative analyses of human samples. To this end, the 295 serum samples tested positive by both assays were used to determine the correlation. We found a good linear correlation between the two assays (R^2^ = 0.946, p < 0.001) (Fig. 3D). The fitted regression line is presented by the equation PBNA = 0.998 × RTTIT−0.019, with 95% CI for the estimated slope found between 0.971 and 1.025 (p < 0.001) and the estimated intercept between-0.060 and 0.022 (p < 0.001). Comparing the PBNA values to the RFFIT values for the samples tested, the average recovery of PBNA was 106% with RSD 26.0%.

To further confirm the correlation between the two assays, we employed Bland–Altman model^31^, To this end, the differences in antibody concentration between each sample (log_10_ IU/ml PBNA−log_10_ IU/ml RFFIT) were plotted against the mean values obtained by the two assays (Fig. 3E). The mean difference was −0.022 log_10_ IU/ml, with SD of the difference being 0.125 log_10_ IU/ml. Based on the Bland–Altman plot, the limits of agreement were –0.271 and 0.228 log_10_ IU/ml (mean ± 2 SD), revealing that the differences for all 295 positive samples pre-determined by both assays fall within the limits of agreement.

Collectively, these data indicate that there is a statistically strong correlation between PBNA and RFFIT in qualitative and quantitative analyses of clinical samples whereas the former is at least 25 times more sensitive.

### Development of *in vivo* PBNA

**Figure 4:**
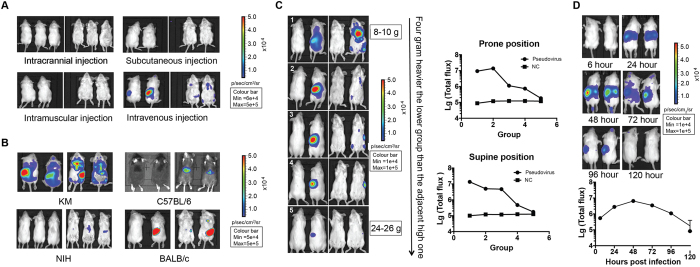
Development of the pseudovirus *in vivo* assay. Panel 4 A: selection of the injection routes. Kunming mice were inoculated with pseudovirus through various routes including intracranial, subcutaneous, intramuscular, and intravenous injections; only intravenous injection resulted in detectable bioluminescence signals. Panel 4B: selection of the mouse lines. When KM, C57BL/6, NIH, and Balb/c were intravenously inoculated with same amount of rabies pseudotyped virus, the highest signals were detected in KM mice. Panel 4 C: selection of the mice weight. KM mice with body weight ranging from 8 to 26 grams were inoculated with 1 × 10^6^ TCID_50_ per mouse. The lighter the body weight of the KM mice, the higher signals could be detected in them Panel 4D: selection of the detection time points. To determine the optimal time points for signal detection, two KM mice were injected with 1 × 10^6^ TCID_50_ pseudovirus and monitored from six hours post-injection to 5 days. By day 2, the intensity of the signals increased and reached the highest level.

To investigate whether the high-titer pseudotyped virus preparations could be used to develop *in vivo* assay for rabies vaccine evaluation, we conducted animal experiments in which mice (Kunming mice, KM) were inoculated with pseudovirus through various routes including intracranial, subcutaneous, intramuscular, and intravenous injections; it turned out that only intravenous injection resulted in detectable bioluminescence signals (Fig. 4A).

We next investigated which murine strain was best suitable for the *in vivo* assay. To achieve this, KM, C57BL/6, NIH, and Balb/c were intravenously inoculated with same amount of rabies pseudotyped virus. As shown in Fig. 4B, the highest signals were detected in KM mice 48 hours post-injection.

We also determine the optimal weight of the KM mice for the *in vivo* assay. KM mice with body weight ranging from 8 to 26 grams were inoculated with 1 × 10^6^ TCID_50_ per mouse. As shown in Fig. 4C, the lighter the body weight of the KM mice, the higher signals could be detected in them following intravenous (*i.v.)* injection. Therefore, we conclude that intravenous inoculation of KM mice weighing 8–10 grams should be chosen to conduct rabies vaccine evaluation using the pseudovirus

Experiments were next conducted to determine the optimal time points for signal detection in the mice following *i.v.* injection. To this end, two KM mice were injected with 1 × 10^6^ TCID_50_ pseudovirus. Six hours post-injection, we started to monitor these animals through bioluminescent imaging for up to 5 days. The bioluminescent signals were visualized in the spleen and liver one day post-inoculation. By day 2, the density of the signals increased and reached the highest level in these organs. The signal density began to decline in these two tissues by day 3, while spread of the pseudovirus to the brain was detected. Three days post-injection, the signal intensity declined rapidly with complete disappearance by day 4, an observation which is largely expected, given that the pseudoviral infection is a single-round event. These findings indicate that 48 h post-infection should be the optimal time point for our *in vivo* assay.

### Comparison of the *in vivo* PBNA with mouse model using wt rabies virus

**Figure 5:**
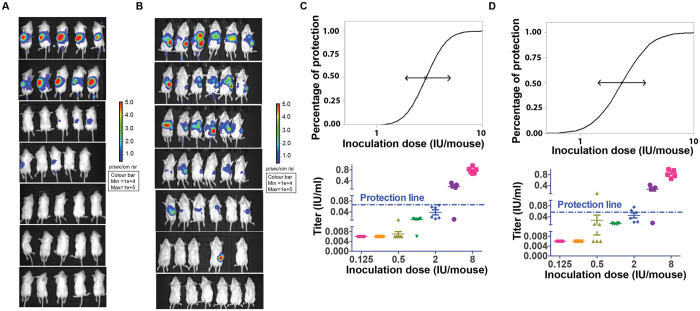
Comparison of the pseudovirus *in vivo* assay with the live wt virus animal model. Panel 5A: selection of the pseudovirus dose used in the *in vivo* assay. To determine the dose for pseudovirus inoculation, serially diluted pseudoviruses were injected intravenously into seven groups of mice (6 mice/group). 50% animal infectious dose (AID_50_) for the pseudovirus was found to be 6.4 × 10^4^ TCID_50_. The pseudovirus dose was determined to be 40 AID_50_ which is equivalent to 2.56 × 10^6^ TCID_50_. Panel 5B:visulization of the protective potency of the HRIGR in pseudovirus *in vivo* assay. Rabies-specific immunoglobulin was injected into KM mice intramuscularly three days before challenge with either wt virus or pseudovirus. A clear dose-response was observed for the pseudovirus *in vivo* assays. Panel 5 C: ED_50_ curve for pseudovirus assay and serum sample antibodies. For the PBNA, the ED_50_ was determined to be 2.89 IU (95% confidence interval: 1.88–4.93 IU) using the probit method. Panel 5D: ED_50_ curve for live virus modeland serum sample antibodies. For the traditional mouse assay using live wt virus, the ED_50_ was 2.31 IU (95% CI: 1.43–3.75 IU).

Finally, we compared our *in vivo* pseudovirus assay with the traditional mouse assay employing wt rabies virus. We first determined the dose for pseudovirus inoculation. To this end, serially diluted pseudoviruses were injected intravenously into seven groups of mice (6 mice/group); the starting dose for each animal was 2 × 10^3^ TCID_50_ as predetermined by PBNA. As shown in Fig. 5A, 50% animal infectious dose (AID_50_) for the pseudovirus was found to be 6.4 × 10^4^ TCID_50_, while for the *in vivo* protective assay, the pseudovirus dose was determined to be 40 AID_50_ which is equivalent to 2.56 × 10^6^ TCID_50_.

We next investigated the correlation between *in vivo* PBNA and the current live virus-based mouse assay. Towards this end, we injected rabies-specific immunoglobulin into KM mice intramuscularly three days before challenge with either wt virus or pseudovirus. As shown in Fig. 5B, a clear dose-response was observed for the pseudovirus *in vivo* assays; the ED_50_ was determined to be 2.89 IU (95% confidence interval: 1.88–4.93 IU) using the probit method(Fig. 5C). For the traditional mouse assay using live wt virus, as shown in Fig. 5D, the ED_50_ was 2.31 IU (95% CI: 1.43-3.75 IU).

As further confirmation, serum samples prior to the virus challenge were collected and tested for their *in vitro* neutralization activities using the PBNA. It was found that serum samples from the pseudovirus *in vivo* assay showed complete protection with neutralizing antibody concentration at 0.65 IU/ml or higher. Two animals were not completely protected, with neutralization titers found to be between 0.55 and 0.65 IU/ml. For the live virus mouse model, full protection could be achieved with the neutralizing antibody level at 0.55 IU/ml or greater. Collectively, these data indicate that the pseudovirus *in vivo* assay can be used to assess the effectiveness of anti-rabies antibodies.

## Discussion

Currently, both *in vitro* and *in vivo* assays for the rabies vaccines and therapeutic rabies-specific immunoglobulin require the use of live wt rabies virus which must be handled at level 2 biocontainment facility. In addition, the *in vivo* assay takes at least two weeks and is known to be labor intensive. Clearly, exploring alternative methods should facilitate the development and evaluation of rabies vaccines and therapeutic antibodies.

Pseudovirus-based neutralizing assays (PBNA) offer great advantages over the wt virus-based methods because they are versatile and much safer to handle. The versatility of pseudovirus is achieved by pseudotyping the virus with different outer membrane proteins or envelope proteins, enabling them to infect a variety of cell types. Pseudovirus is much safer because the virus is essentially devoid of virulent viral components and involves in a single round of replication. Yet, the biggest drawback of pseudovirus system is that the harvested viruses are often of very low titers, substantially limiting their applications, particularly for *in vivo* studies. We resolved this issue by systematically analyzed the type of promoter, various combinations of rabies-G protein expressing plasmid and the core plasmid expressing the luciferase in addition to optimization of all critical steps in high-yield pseudovirus production.

With respect to reproducibility of *in vitro* assay, PBNA is also more reproducible than the traditional RFFIT assay as demonstrated by smaller difference in values assigned to the two internal control samples and smaller CV values compared with those assigned by RFFIT. When the serum titers were calculated against the national standard, we observed a good agreement in values between the two assays; however, PBNA is more objective and less labor intensive as the data are were obtained through luminescent reading, while the operator of RFFIT have to read the results manually under the microscope.

The biodistribution of the pseudovirus in mice were systematically investigated. Given that spleen and liver are the major organs clearing particles in blood circulation, the biodistribution pattern of the rabies pseudovirus administered intravenously is expectedly to be different from that of the wt virus model in which the virus is given intramuscularly^32^; in the latter case, the rabies virus reaches the CNS by retrograde transport route, followed by spreading centrifugally along the autonomic and sensory nerves to the peripheral organs^32^. In our pseudovirus model, the bioluminescence was found in brain by 72 hours, a time at which the signal density began to decline in spleen and liver, suggesting that the pseudovirus maintains neurotropic property. Nevertheless, as the spleen is known to be the biggest reservoir for monocytes that outnumbered those in circulation and both human and mouse monocytes are susceptible to rabies viral infection^33^, this could also explain the strong luminescence signal in the spleen. As expected, after we intracerebrally injected the animals with pseudovirus, the bioluminescent signal was initially found to be mainly localized in the brain, followed by re-distribution into the spleen and liver, which is a biodistribution pattern similar to that of the live wt virus given intracerebrally (data not shown). Therefore, the difference in viral biodistribution is largely due to route of administration. Despite of the different viral distribution^22^,^34^,^35^, data generated with the PBNA *in vivo* assays demonstrated strong statistical correlation with those obtained with the live wt virus-based assay.

In short, both PBNA *in vitro* and *in vivo* assays are superior to the wt virus-based assays. Specifically, the absence of lethal wt virus in the whole experimental procedure could greatly facilitate the development of rabies vaccine and therapeutics; in addition, the pseudovirus *in vitro* assay is more sensitive and reproducible as well as less labor-intensive compared to the traditional assay. Moreover, PBNA *in vivo* assay shortened experimental time from 2 weeks to just 3 days. Nonetheless, our method for the preparation of high-titer pseudovirus and optimization of *in vitro* and *in vivo* assays could be of interest to vaccine developers and regulators alike.

## Methods

### Cells, viruses, and serum samples

HEK293 (ATCC, CRL1573), 293T(ATCC, CRL3216), 293FT(Invitrogen, Carlsbad, CA, USA), MDCK(ATCC, CCL34), BHK21 (ATCC, CCL10), BSR (from Institut Pasteur, Paris, France) and Vero (ATCC, CCL81) cells were maintained in a 5% CO_2_ environment at 37 °C in high glucose DMEM (GIBCO) supplemented with 10% FBS (GIBCO), penicillin (100 IU/ml), and streptomycin (100 μg/ml), and passaged every 2–3 days.

RABV challenge standard CVS-11 was kindly provided by Institut Pasteur, Paris, France, and passaged in BSR to establish the primary, master, and working virus seed lots. The working seed were used in the RFFIT and live virus animal model.

50 rabies-negative human serum samples were kindly provided by Shanghai RAAS Blood Products Co. Ltd (Shanghai, China)^36^ for the specificity determination of the rabies PBNA. 320 post-vaccinated human serum samples were collected from a phase III clinical trial for a rabies vaccine produced in Vero cell (chinadrugtrials.gov.cn ID: CTR20140821) were kindly provided by Hualan Biological Engineering, Inc. (Henan, China). Healthy volunteers received rabies vaccination intramuscularly on day 0 (2 doses), 7 (1 dose), and 21 (1 dose). Of the 320 serum samples, 32 were collected on day 7 before the second vaccination, while 288 were collected on day 35. The national standard for anti-rabies immunoglobulin collected from post-vaccination individual was established and calibrated with the 2nd International Standard for anti-rabies immunoglobulin, human (NIBSC code: RAI). Two serum samples used as the internal control for the RFFIT were also collected from post-vaccinated individuals and calibrated using the International standard. The assigned value for the national standard was 37.0 IU/ml. The assigned values for the internal control samples A and B were 2.2–3.6 IU/ml and 7.1–12.9 IU/ml (average ± SD) respectively. Written informed consents were obtained from all the volunteers.

### Construction of Fluc, G protein and HIV-1 backbone expressing plasmids

Firefly luciferase (*fluc*) gene was cloned from pCLucf^37^, a gift from John Schiller (Addgene plasmid # 37328) and subsequently inserted into the BamH I-Xho I sites of the pcDNA3.1 + (Invitrogen, Carlsbad, CA) to generate plasmid pcDNA3.1.fluc. Promoters full-length CMV (pDRV1.0 kindly provided by Yiming Shao, China CDC), CAG (pCAGGS^38^, a kind gift from Yuelei Shen, Biocytogen Co., Ltd, China), and LTR (pSG3ΔEnv^39^, kindly provided by Xiaoyun Wu, University of Alabama) were used to replace the CMV promoter in pcDNA3.1 + through Mlu I-Nhe I digestion and in-fusion ligation (clontech) to generate psCMV.fluc, pCAG.fluc, and pLTR.fluc respectively. The *fluc* genes in the *fluc*-expressing plasmids were replaced by G protein genes from CVS-11 by BamH I-Xho I digestion to generate the pcDNA3.1.CVSG, psCMV.CVSG, pCAG.CVSG, and pLTR.CVSG respectively.

Fluc from pcDNA3.1.fluc and sCMV.fluc from psCMV.fluc were amplified and cloned respectively into pSG3ΔEnv to generate pSG3ΔEnv.fluc using restriction endonuclease digestion (Hpa I) and direct ligation (In-Fusion). Similarly, sCMV-fluc was constructed to generate pSG3ΔEnv.sCMV.fluc. All the primers used in this section were listed in Table 1.

### Production and titration of pseudoviruses

Production of rabies pseudoviruses in mammalian cells was accomplished using methods similar to HIV pseudovirus described previously^28^,^40^. Briefly, mammalian cells were cotransfected with rabies G protein expression plasmids and the HIV backbone vector using Lipofectamine 2000 (Invitrogen, Carlsbad, CA) according to the manufacturer’s instruction. Forty-eight hours post transfection, pseudovirus-containing culture supernatants were harvested, filtered (0.45-μm pore size) and stored at −70 °C or lower in 1-ml aliquots until use. 50% tissue culture infectious dose (TCID_50_) of each rabies pseudovirus batch was determined using a single-use aliquot from the pseudovirus bank; all stock was used only once to avoid inconsistence which could have resulted from repeated freezing-thawing cycle. Serial 5- fold dilutions (9 dilutions in total) were made in hexaplicate wells of 96-well culture plates with a 50-fold initial dilution. The last column was designated as the negative control without pseudovirus. Trypsin-treated mammalian cells adjusted to a concentration mixed with DEAE-dextran were seeded to each well. After 48 h incubation in a 5%CO_2_ environment at 37 °C, culture medium was aspirated gently to leave 100 μl in each well; subsequently,100 μl of Bright-Glo luciferase reagent (Promega, Madison, WI) was added to each well. Following 2 min incubation at room temperature, 150μl of lysate was transferred to solid black 96-well plates for luminescence detection using Glomax 96 microplate luminometer (Promega, Fitchburg, WI). The TCID_50_ was calculated using the Reed-Muench method^41^.

### *In vitro* PBNA

Neutralization was measured by the reduction of the *luc* gene expression as described previously in HIV pseudovirus neutralization assay^40^,^42^. 50% inhibitory concentration (IC_50_) was defined as the serum dilution at which the relative light unit (RLU) was reduced by 50% compared with virus control wells (virus + cells) after subtraction of the background RLU in control groups with cells only. In brief, pseudovirus was incubated with serial dilutions of test samples (8 dilutions in a 3-fold step-wise manner) in duplicate for 1 hour at 37 °C, together with virus control and cell control wells in hexaplicate. After that, freshly trypsinized cells were added to each well. Following 48 hours of incubation in a 5%CO_2_ environment at 37 °C, the luminescence was measured as described in the Section for pseudovirus titration. The IC_50_ values were calculated with non-linear regression, i.e., log (inhibitor) vs. response (four parameter), using GraphPad Prism 6 (GraphPad Software, Inc., San Diego, CA). The neutralizing antibody titer was calculated by comparing with the national anti-rabies standard (37.0 IU/ml). Samples with neutralizing antibody titer equal or more than 0.5 IU/ml was defined as adequate for protection against rabies^43^; they were therefore determined as positive.

### Rapid fluorescent focus inhibition test (RFFIT)

Neutralizing antibody titers against genuine rabies virus were determined by RFFIT as described previously^44^. Briefly, 50 μl CVS-11 (20000 FFU_50_/well) was incubated with 100 μl serial dilutions of test samples (8 dilutions in a 3-fold step-wise manner) in duplicate for 1 hour at 37 °C. Next, 50 μl BSR cells (1 × 10^6^/ml) were added to each well and incubated in a 5%CO_2_ environment at 37 °C for 24 h. Finally, cells were fixed with chilled 80% acetone at 4 °C for 30 min and stained with FITC-conjugated anti-rabies N monoclonal antibody (Fujirebio Diagnostics, Inc., Malvern, PA) at 37 °C for 30 min. Recorded the fluorescent rate per well visually under a fluorescence microscope. Average infection rate of duplicate samples was then determined. The neutralizing antibody titers were determined by comparing with the national standard using the calculating method reported previously^44^. For each test, the internal control samples A and B were included to ensure the effectiveness of the assay, in which both of the values for A and B should lie in the pre-determined ranges respectively.

### Animal experiments

All mice were housed and maintained in accordance with the relevant national guidelines and regulations. All procedures were carried out according to the protocols approved by Institutional Animal Care and Use Committee of the National Institute for Food and Drug Control (NIFDC). All animals were obtained from Institute for Laboratory Animal Resources, NIFDC. The mice were inoculated with human rabies immunoglubins (HRIG) intramuscularly three days before virus challenge and bled just before virus challenge. The neutralizing titers of the mouse serum samples were determined using rabies PBNA. For the pseudovirus*in vivo* challenge assay, the mice were inoculated with different volumes for pseudoviruses, i.e., 0.1 ml for intramuscularly, 0.03 ml for intracerebrally, and 0.5 ml for subcutaneously and intravenously. For the wt virus challenge assay, the mice were inoculated with CVS-11 with a volume of 0.1 ml (40 LD_50_) intramuscularly. For the pseudovirus challenge assay, bioluminescence was detected for each mouse. For the wt virus challenge, survival rates or signs of rabies were recorded for each group during a time period between 5 and 14 days after challenge.

### *In vivo* bioluminescence imaging analysis

Bioluminescence analyses was conducted using IVIS-Lumina III imaging system (Xenogen, Baltimore, MD) as described previously^45^,^46^. Briefly, mice were anesthetized by intraperitoneal (i.p.) injection of pentobarbital sodium (40 mg/kg body weight), followed by an i.p. injection of the substrate D-luciferin (150μg/g body weight, Xenogen-Caliper Corp., Alameda, CA). Seven minutes later, bioluminescence was detected for each mouse in the imaging chamber with an acquisition time of 1 min. The relative bioluminescence was calculated using a photon-per-second mode with normalization for the imaging area (photons/s/cm^2^/sr) (total flux) as previously described^47^.

### Statistical analysis

One way analysis of variance was used to assess the difference of the pseudovirus titers generated in various cell lines or in different cell numbers. The paired comparisons were also conducted using student’s t test. A paired *x*^*2*^ test (McNemar’s *x*^*2*^test) and kappa values were used to assess the difference in qualitative results obtained from PBNA and RFFIT. Pearson’s correlation coefficient was employed to analyze the strength of the linearity between the log_10_-transformed values for PBNA and RFFIT. To compare the quantitative results obtained for the positive samples detected by both assays, a fitted regression model was compared by testing the two- tailed hypothesis of slope 1 and intercept 0. Bland-Altman method, i.e., a scatter plot of the differences between the paired measurements against the mean values of the samples, was used to assess the magnitude of disagreement between the two assays^31^,^48^. All graphs were generated using Prism 6.0c software (GraphPad, San Diego, CA).

## Additional Information

**How to cite this article**: Nie, J. *et al*. Development of *in vitro* and *in vivo* rabies virus neutralization assays based on a high-titer pseudovirus system. *Sci. Rep.* **7**, 42769; doi: 10.1038/srep42769 (2017).

**Publisher's note:** Springer Nature remains neutral with regard to jurisdictional claims in published maps and institutional affiliations.

## Figures and Tables

**Table 1:** Primers for the plasmid construction.

Primer code	Primer sequence
FlucF	GCCACCATGGAAGATGCCAAAAACAT
FlucR	TTATTACACGGCGATCTTGCCGCCCT
CAGF	GGCCAGATATACGCGCTAGTTATTAATAGTAATCAATTACG
CAGR	AAGTTTAAACGCTAGAATTCTTTGCCAAAATGATGAGAC
CMVF	GGCCAGATATACGCGACCGCCATGTTGACATTGATT
CMVR	AAGTTTAAACGCTAGCGTGTCGACGACGGTGACTGC
LTRF	GGCCAGATATACGCG GCAGTATCTCGAGACCTAGAA
LTRR	AAGTTTAAACGCTAGTACCTCCTGGGTGCTAGAGA
CVS-11OF	TTGAGCCTCTTGGATGTG
CVS-11OR	TCGTCAAAAGGATGACCG
CVS-11IF	TACCGAGCTCGGATCGCCACCATGGTTCCTCAGGTTCTT
CVS-11IR	GCCCTCTAGACTCGATCACAGTCTGATCTCACCTCCACTCCTATATGA
Fluc-F-HpaI	AAGAATAGTGCTGTTGCCACCATGGAAGATGCCAAAAACAT
Fluc-R-HpaI	GACATTAAGCAAGTTTTATTACACGGCGATCTTGCCGCCCT
cmvFluc-F-HpaI	AAGAATAGTGCTGTTTGCTTCGCGATGTACGGGCCAGA
